# Relationship Between Depressive Symptoms and Quality of Life in Family Caregivers of Dependent Children: A Systematic Review with Meta-Analysis

**DOI:** 10.3390/healthcare14172765

**Published:** 2026-09-01

**Authors:** Francisco Segura-Galán, Catalina López-Martínez, Belen Gutiérrez-Sánchez, Rafael del-Pino-Casado

**Affiliations:** Nursing Department, Universidad de Jaén, 23071 Jaén, Spain; fsegura@ujaen.es (F.S.-G.); cmartine@ujaen.es (C.L.-M.)

**Keywords:** family caregivers, depressive symptoms, quality of life, children

## Abstract

**Background:** Caring for dependent children with chronic illnesses or disabilities constitutes a major public health challenge, subjecting family caregivers to prolonged physical and emotional stressors. Objectives: To quantitatively synthesize the relationship between depressive symptomatology and quality of life (QoL) in this population. **Methods:** A systematic review methodology with meta-analysis was conducted following PRISMA guidelines and Cochrane handbook recommendations. Databases such as PubMed, CINAHL, PsycInfo and Scopus were consulted up to February 2026. **Results:** Thirty-three original studies evaluating family caregivers of children under 18 years of age and reporting statistical data of association were included. The meta-analysis revealed a statistically significant moderate-to-strong negative association between depressive symptoms and overall QoL (r = −0.532; I^2^ = 9.51%), maintaining similar values across all analyzed domains (physical, mental, social and environmental) as well as the summary components of the SF-36 questionnaire. According to the GRADE criteria, the overall certainty of evidence was rated as low to very low across all QoL domains and SF-36 components. **Conclusions:** This study demonstrates that heightened depressive symptomatology is substantially associated with compromised well-being among family caregivers.

## 1. Introduction

Providing care for children presenting chronic diseases, disabilities, or developmental disorders represents a multifaceted public health challenge that extends beyond strictly clinical and familial boundaries [1]. Assuming the role of a family caregiver for a dependent child entails prolonged exposure to physical, emotional, and financial burdens that frequently exceed individual resources and available coping strategies [2]. Consequently, caregivers often find themselves in a state of structural vulnerability, wherein continuous devotion to the child’s needs occurs to the detriment of the adult’s self-care and overall well-being [3].

This scenario can be understood through two fundamental theoretical frameworks. On the one hand, the Lazarus and Folkman Model posits that caregiver distress arises when the perceived demands of the child’s condition outweigh the caregiver’s cognitive resources and coping strategies [2]. On the other hand, Pearlin’s Stress Process Model provides a sociological vision, contending that chronic caregiving acts as a primary stressor that proliferates into secondary domains, systematically eroding the individual’s self-concept, economic stability, and social support network [3]. Both models illustrate how caregiver vulnerability is mediated by both subjective appraisal and structural attrition.

The extant literature indicates a higher prevalence of depressive symptomatology among caregivers of dependent children compared with the general population [4]. Chronic stressors such as social isolation, persistent uncertainty surrounding the child’s disease trajectory and prognosis, role overload, and the disruption of personal life projects constitute significant risk factors for the onset of affective disorders [5]. The manifestation of depressive symptoms in caregivers not only undermines their mental health but may also adversely affect the quality of care provided, treatment adherence, and the integrity of the caregiver–child bond [6].

Concurrently, QoL has emerged as a cornerstone construct for evaluating the broader impact of prolonged care from a multidimensional standpoint [7,8,9]. Encompassing physical, psychological, social, and functional domains, QoL offers a holistic vision of caregiver well-being.

In recent years, several systematic reviews and observational studies have analyzed the impact of family caregiving, consistently highlighting high levels of caregiver burden and its deleterious effects on psychological health [5,10]. However, most existing reviews have primarily focused on descriptive summaries of burden or individual psychiatric outcomes separately, rather than quantitatively synthesizing the direct relationship between depressive symptomatology and multidimensional quality of life across diverse pediatric conditions.

Despite the expanding body of empirical research addressing depression and QoL in family caregivers of dependent pediatric populations, the absence of quantitative synthesis restricts the generalizability of findings and hampers the formulation of evidence-based interventions. In this context, the performance of a meta-analysis is methodologically justified to integrate the empirical evidence, quantify the pooled effect size of the association between both constructs, and explore potential sources of heterogeneity through subgroup analyses.

Accordingly, the primary objective of this meta-analysis is to quantitatively synthesize existing evidence regarding the relationship between depressive symptoms and quality of life in family caregivers of dependent children.

## 2. Methodology

### 2.1. Design

A quantitative systematic review with meta-analysis was conducted in accordance with the recommendations of PRISMA [11] and the Cochrane Handbook [12].

The study protocol was progressively registered in the PROSPERO database [13] under registration ID CRD420251274745, with no deviations or methodological modifications to the original design.

### 2.2. Search Strategy

A comprehensive systematic search was conducted across different international databases, namely, PubMed, CINAHL, PsycInfo, Scopus, from their respective inception dates through February 2026. Table 1 details the search strings deployed across the database platforms.

### 2.3. Eligibility Criteria

Studies were selected based on the following inclusion criteria: (1) original research studies; (2) reports on the relationship between depressive symptoms and quality of life; (3) target population comprising informal caregivers; (4) care recipients defined as dependent individuals under 18 years of age; (5) statistical data on the magnitude of the association between depressive symptoms and quality of life.

To ensure maximum process transparency and replicability, explicit operational descriptions of the title/abstract screening process and full-text evaluation and the exact numbers of articles included and excluded in each phase of the PRISMA flow chart are meticulously detailed at the beginning of Section 3, along with a corresponding explanatory diagram.

### 2.4. Data Extraction

Data were extracted independently by two reviewers (BGS and FSG) using a standardized form (resolving discrepancies through direct consensus with an inter-rater agreement of 96.5%).

The following data were collected: author and publication year, study design, sample size, average age of family caregivers of the child/adolescent, duration of care, average age of the minor, caregiving duration, percentage of female caregivers, percentage of caregiver spouses, depressive symptoms scale, QoL scale, primary diagnosis of the dependent care recipient, and association size.

### 2.5. Quality Assessment of the Included Studies

Risk of bias in primary studies was evaluated using the domain-based assessment toll developed by the Agency for Healthcare Research and Quality (AHRQ) [14] alongside the analytical criteria proposed by Boyle [15]. A domain-specific approach was chosen over summary score scales to enable a domain-specific, non-quantitative assessment of core sources of bias. The evaluation criteria included: (1) the type of sampling for selection bias and (2) the validity and reliability of the instruments for classification bias, (3) controlling for variables such as support network size, age, and sex for confounding bias. Effective confounding required matching, stratification, or multivariate analytical procedures. In multivariate analyses, the absence of bias was established if the variation between unadjusted and adjusted effect sizes remained under 10%, according to Rothman et al. [16].

Quality evaluations were conducted independently by two reviewers (BGS and FSG) with a percentage of agreement of 90%.

### 2.6. Assessing the Quality of the Evidence

Following the recommendations proposed by GRADE (Grading of Recommendations Assessment, Development and Evaluation) [17], the quality of the evidence was determined from several key domains: the methodological robustness of the included studies (previously described), the presence of inconsistency results, the degree of imprecision, and possible publication bias. Overall, the certainty of evidence was downgraded to low or very low across outcomes, primarily due to serious risk of bias stemming from cross-sectional designs, non-probabilistic sampling, and unadjusted residual confounding in the primary studies (Supplementary Material S1).

Inconsistency was evaluated by inspecting variability in effect estimates across studies while accounting for sampling variance. Imprecision was examined based on the impact of sample size, assessed through the breadth of confidence intervals, the number of participants, and the number of events recorded. Similarly, publication bias, which is understood as the probability of the existence of non-disseminated studies, was analyzed by inspecting funnel charts and the statistical tests described in Section 2.7.

Publication bias was assessed via visual inspection of funnel plots and statistical tests, as detailed in Section 2.7. For consistency evaluations, studies were categorized by count (small (<5), intermediate (5–10), or sufficient (>10)) and by mean sample size (small (<100), moderate (100–300), or large (>300)).

### 2.7. Analysis

To accommodate the generalizability of the findings to diverse informal caregiver populations, meta-analyses were implemented using random-effects models [18]. Pearson correlation coefficients (r) served as the primary effect size metric. To ensure data independence and satisfy model assumptions, a single effect size was included per independent study population. Inter-study heterogeneity was quantified via the Cochran Q test (assuming no heterogeneity with a *p*-value > 0.10) and I^2^ statistic [19]. The latter allowed the variability to be categorized as low (25%), moderate (50%), or high (75%). On the other hand, publication bias was assessed following Guyatt et al. [20] by means of various procedures, i.e., inspection of the funnel diagram (funnel plots), the Egger regression test [21], and the Trim and Fill technique [22] for the estimation of the effect size in a scenario absent publication bias.

To ensure data independence and avoid bias from duplicate sampling, it was ensured that no population contributed more than one effect size to the same meta-analytic model. When primary studies assessed multiple dimensions or components of quality of life (i.e., the physical and mental components of the SF-36 or SF-12 Health Questionnaire, or the physical, social, mental, and environmental subdimensions), these were not combined simultaneously into a single overall effect size. Instead, separate meta-analyses were performed for each dimension of quality of life (overall, physical component, mental component, and specific subdimensions). Similarly, when a study provided disaggregated data for independent subgroups (mothers and fathers), these were treated as separate sampling units in the corresponding analyses. Finally, the robustness of the results was assessed through sensitivity analyses with sequential leave-one-out and subgroup analyses based on quality criteria.

All statistical analyses were executed in Comprehensive Meta-Analysis v.3.3 software.

## 3. Results

### 3.1. Description of the Search Results

**Figure 1:**
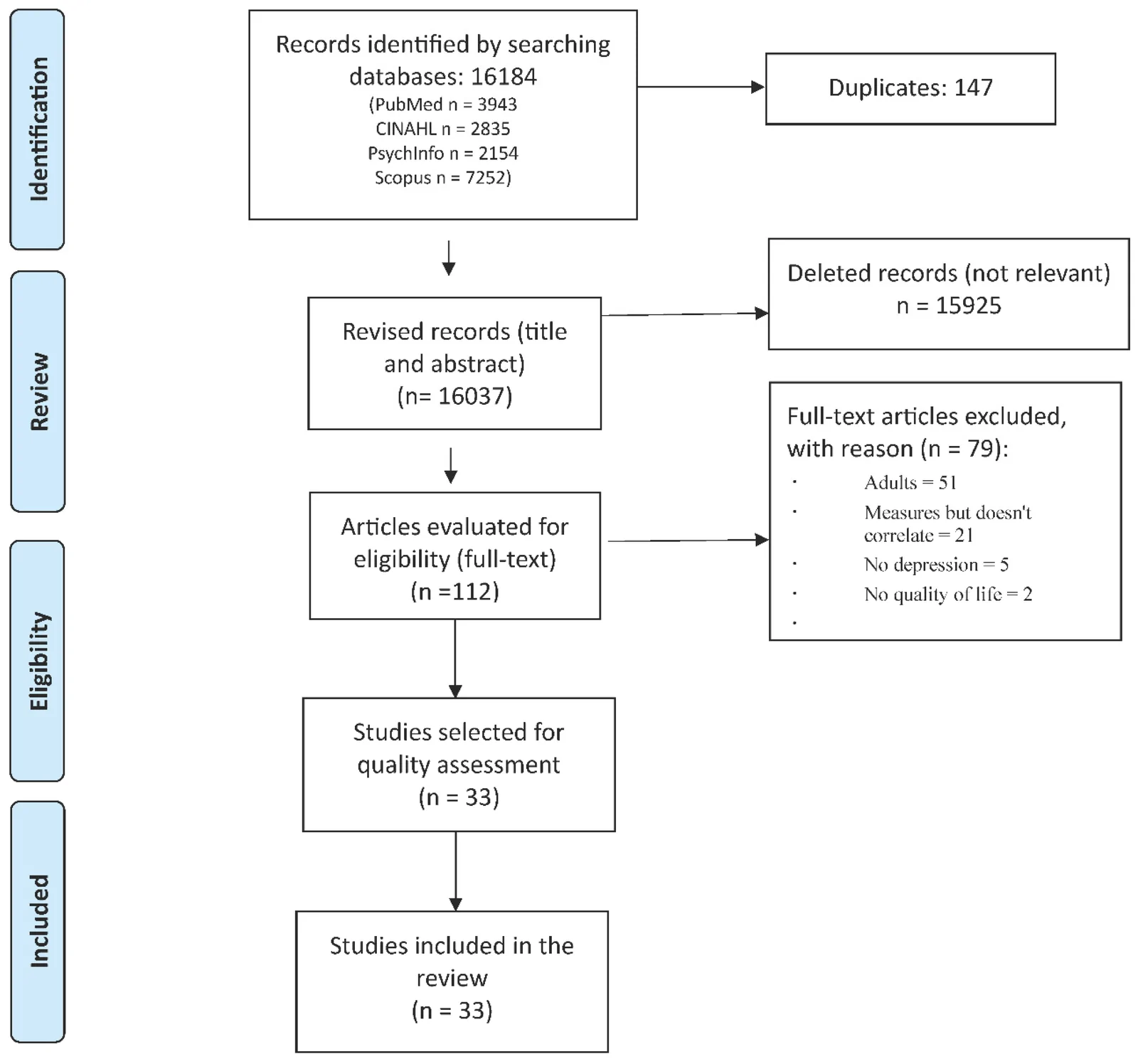
Flowchart.

Database searches yielded an initial total of 16,184 records (Figure 1). Following the removal of 147 duplicates, 16,037 unique articles were obtained for the initial screening, resulting in 15,925 irrelevant studies. Subsequently, 112 full-text articles were evaluated, of which 79 failed to satisfy the inclusion criteria. Ultimately, 33 original studies met all eligibility criteria and were incorporated into the meta-analysis.

### 3.2. Description of the Characteristics of the Included Studies

The analyzed studies demonstrated broad geographical diversity, covering countries in the Americas (USA, Mexico, Colombia, Brazil, Canada), Europe (Germany, Poland, Serbia), Asia (Jordan, Turkey, Bangladesh, Iran, Republic of Korea, Qatar, China, India), Africa (Ghana), and Oceania (Australia). Regarding methodological design, nearly all were cross-sectional descriptive studies, except for one longitudinal study employing repeated measures [23].

Sample sizes ranged from 20 [24] to 579 [25] participants (Table 2). Primary caregivers were predominantly mothers and fathers, though select studies included other relatives. The care recipients comprised children and adolescents with autism spectrum disorder (ASD), neuromotor or neuromuscular conditions, chronic diseases, and Down syndrome.

Depressive symptoms were most commonly measured using BDI or BDI-II, followed by the DASS-21, PHQ-9, and CES-D scales. Quality of life was predominantly assessed using the WHOQOL-BREF and the SF family of questionnaires (SF-36, SF-12, SF-6D), and specialized scales such as CQOLCF, NHP-1, PACQLQ, CQLI-R, Beach Center FQOL Scale, and EQ-5D were also applied.

### 3.3. Description of the Quality of the Included Studies

The analysis of the risk of bias of the 33 included studies (Table 3) reveals a consistent pattern in the dimensions of selection, classification, and confounding. Concerning selection bias, all studies presented non-probability sampling. On the contrary, absolute methodological strength was observed in classification bias, where 100% of the studies satisfactorily complied with the categorization and measurement of exposure and outcome variables.

Concerning confounding bias, 90.9% (n = 30) of the studies did not report effective control of confounding variables. However, three works [26,31,51] distinguished themselves by integrating appropriate adjustments or controls against these factors. Finally, in four studies [23,30,44,53], this bias could not be assessed due to lack of data.

### 3.4. Description of the Results of the Meta-Analysis

The results of the different meta-analyses are shown in Table 4 (see also Supplementary Material S2).

### 3.5. Global Quality of Life

**Figure 2:**
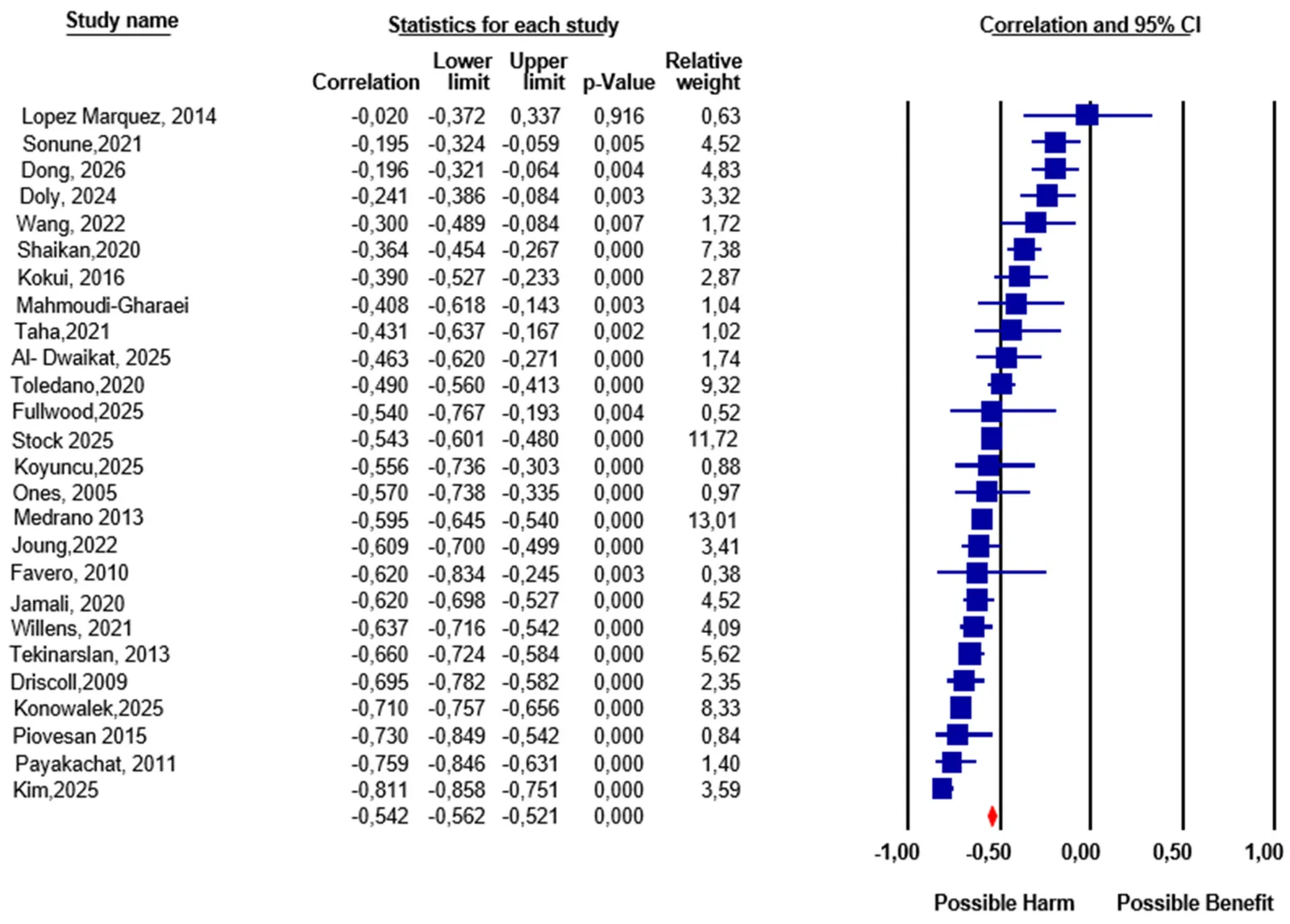
Forest plot showing the relationship between depressive symptoms and quality of life in caregivers of dependent children. Note: The red diamond represents the total effect size of the meta-analysis. The studies in the figure correspond to references [23,24,25,26,27,28,29,30,31,32,33,34,35,36,37,38,39,40,41,42,43,44,45,46,47,48,49,50,51,52,53,54,55].

**Figure 3:**
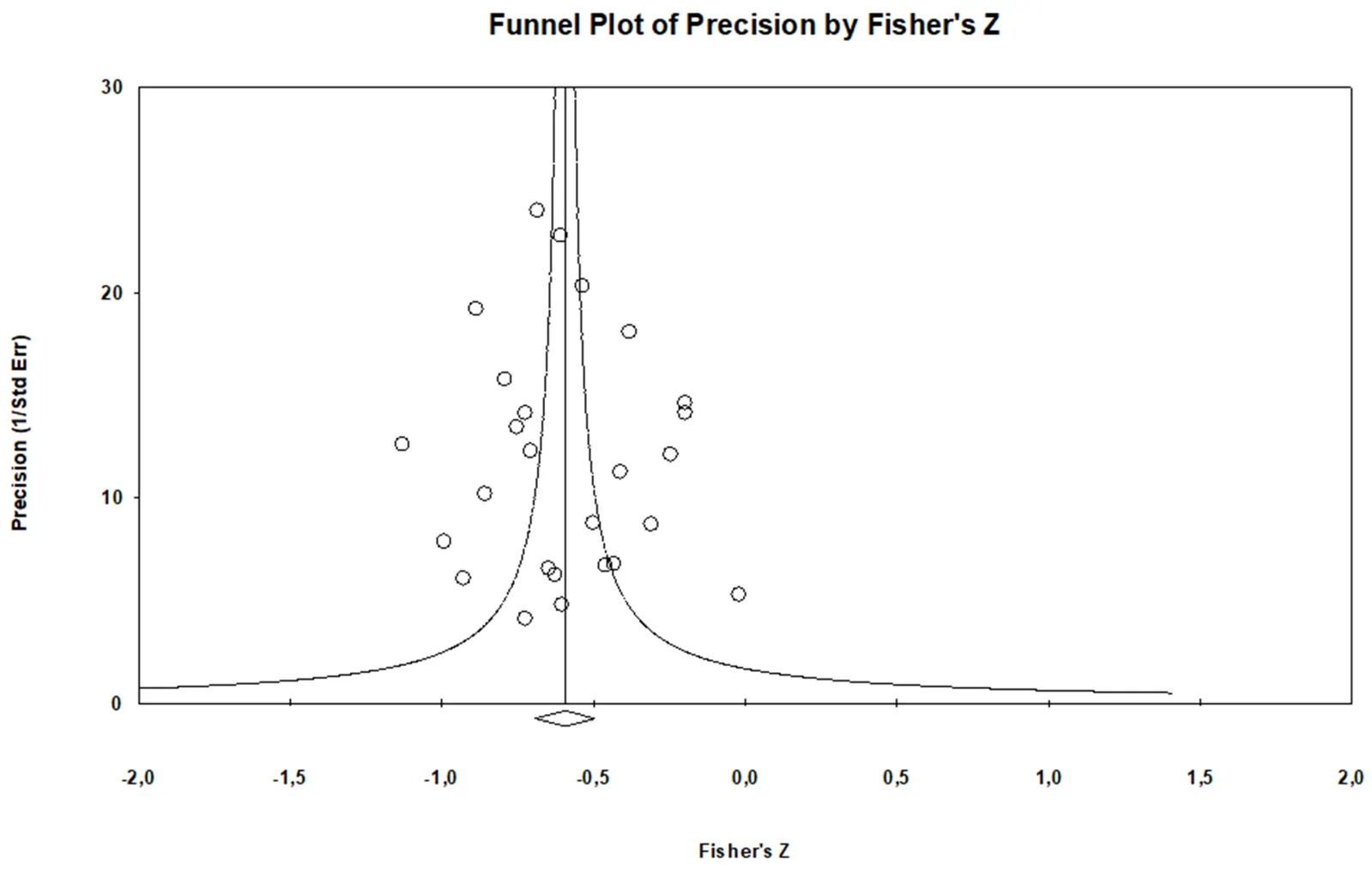
Funnel plot of the relationship between depressive symptoms and quality of life of family caregivers of dependent children.

Meta-analysis of twenty-six studies demonstrated a statistically significant, moderate-to-strong negative association (Figure 2) between depressive symptoms and overall QoL (correlation coefficient (r = −0.532; 95% CI = −0.599; −0.458; N = 4238; average N = 163)). Regarding the heterogeneity of the results, an I^2^ value of 9.51% and a Q statistic of 27.62 (*p* = 0.32) were found. These values indicate that the variability observed between studies is low. Regarding publication bias, the analysis of the funnel graph (Figure 3) showed asymmetry, although the Egger test presented a value of 0.73. Furthermore, the Trim and Fill procedure yielded an unadjusted correlation of −0.532, supporting the stability of the observed effect.

Descriptive analysis by subgroups according to the cause of dependency (Table 4) revealed statistically significant associations in all dimensions evaluated. These findings suggest, in an exploratory manner, that the association between the variables remains present across the different populations of caregivers studied.

Consistent with the quality criterion of control for confounding variables, correlation coefficients maintained their significance and a similar intensity, suggesting stability in the observed relationships.

### 3.6. Analysis of the Components of the SF-36 (PCS and MCS)

When quality of life was disaggregated into the component scores of the SF-36 questionnaire, similar effects were observed for both the physical summary component (PCS) and the mental summary component (MCS). Seven studies using this questionnaire were identified. For the PCS, the pooled analysis revealed a statistically significant negative association with a strong overall effect size (r = −0.544; 95% CI = −0.727; −0.289; N = 503; N mean = 71.86). Similarly, the MCS showed a statistically significant negative association with a strong pooled effect size (r = −0.537; 95% CI = −0.717; −0.290; N = 503; average N = 71.86).

### 3.7. Quality of Life Analysis Dimensions

Studies assessing the different dimensions of quality of life (environmental, physical, psychological, and social) were also examined. We identified nine studies for the first three dimensions and eight for the social dimension. A negative association with a moderate-to-strong effect size in all dimensions for environmental quality of life (r = −0.410; 95% CI = −0.518; −0.289), physical quality of life (r = −0.481; 95% CI = −0.587; −0. 358), psychological quality of life (r = −0.527; 95% CI = −0. 635; −0. 399), and quality of social life (r = −0.486; 95% CI = −0.604; −0.347) was observed.

## 4. Discussion

The primary finding of this meta-analysis reveals a robust negative association between depressive symptoms and overall quality of life among family caregivers of dependent children, a consistent pattern observed across all evaluated dimensions and components. Rather than implying a direct causal trajectory, these results underscore a strong co-occurrence between depressive affectivity and lower perceived well-being, suggesting that emotional distress closely mirrors the erosion of global functional resources in this population.

Similarly, within the framework of the Lazarus and Folkman Model [2], depressive symptomatology is identified as a relevant correlate of altered cognitive appraisal in caregivers. Higher depressive symptoms are associated with lower coping capacities and reduced perceived self-efficacy. This offers a plausible explanation as to why, when facing similar levels of patient dependence, caregivers reporting greater depressive symptoms also exhibit significantly lower quality of life, likely reflecting a negative bias in the appraisal of their living conditions.

To date, no prior meta-analyses have quantitatively synthesized the association between depression and QoL specifically within pediatric family caregiving cohorts. However, secondary evidence corroborates that caregiving-related psychological burdens rarely occur in isolation. In this sense, del-Pino-Casado et al. [56] have already indicated strong associations between subjective overload and anxiety. The convergence of these psychological symptoms creates a scenario of vulnerability where depression exhibits a strong statistical relationship with overall well-being.

Regarding the previous literature, caregivers’ QoL is fundamentally conditioned by psychological strain [57]. Although this study incorporates coping as an explanatory variable, its results coincide with findings pointing to a significant inverse relationship between emotional distress and QoL. In this sense, the results expand the evidence by focusing on caregivers of the dependent pediatric population, a less studied group, showing that depressive symptoms represent a key factor associated with the deterioration of QoL. However, the particularities of childcare, such as parental emotional involvement and the chronicity of the process, may modulate this association in ways that are not applicable to caring for the elderly.

Importantly, although the models of Pearlin and Lazarus and Folkman [2,3] conceptualize the influence of negative affectivity on the assessment of well-being, the findings of this meta-analysis must be interpreted strictly within a non-causal frame. Because the included studies predominantly utilized observational cross-sectional designs, directionality cannot be established, and it is not possible to determine whether depressive symptoms precede a decline in quality of life or vice versa. From a clinical standpoint, depressive symptoms and QoL likely exist within a bidirectional, mutually reinforcing feedback loop: chronic caregiving stress diminishes QoL and heightens vulnerability to depressive symptoms, which in turn further impairs subjective QoL [58,59,60].

The extant literature demonstrates that interventions aimed at caregivers can attenuate depressive symptoms while improving overall quality of life. In this regard, research shows significant benefits of social support-based interventions on depressive symptoms in caregivers [61]. Consistently, other studies [62,63,64,65] have shown that psychoeducational programs, cognitive–behavioral interventions, and strategies based on e-Health contribute to enhancing both psychological well-being and QoL in caregiving populations, reinforcing the importance of implementing this type of approach in this group.

Our findings regarding the clinical burden of depressive symptoms align closely with recent empirical evidence in specific pediatric conditions, such as the work by Barutcu et al. [66], who demonstrated high levels of depression, anxiety, and burden among caregivers of children with cerebral palsy. Furthermore, when systematically comparing our primary pooled effect sizes with prior meta-analyses in pediatric caregiving, the inverse magnitude between depression and quality of life remains consistently stronger than those reported for broader health-related outcomes, confirming that affective distress is a primary driver of functional impairment in family caregivers.

### 4.1. Limitations

Several methodological limitations warrant consideration. First, the observational cross-sectional nature of primary studies precludes causal inferences. Second, the majority of primary studies relied on non-probabilistic convenience samples and lacked control for key confounding variables, though leave-one-out sensitivity analyses confirmed the stability of the pooled effect size. Third, heterogeneity in measurement scales across primary studies represents an inherent limitation, despite domain-specific harmonizations. Fourth, the assessment of publication bias has methodological limitations. In accordance with the guidelines established by the Cochrane Handbook [12], the formal test for publication bias was deliberately omitted for subgroup analyses and subdimensions with fewer than 10 studies (K < 10) due to low statistical power.

### 4.2. Implications of Clinical Practice

From a clinical perspective, the robust association observed between caregiver depressive symptoms and reduced quality of life highlights important considerations for family dynamics. In clinical contexts, depressive symptomatology is frequently linked to sleep disruptions and chronic fatigue, which are closely associated with diminished daily energy reserves and altered emotional regulation. These co-occurring psychological burdens may be accompanied by challenges in parental sensitivity and caregiver–child interactions, potentially coinciding with broader effects on the child’s socio-emotional context.

Furthermore, high levels of depressive distress have been observationally correlated with difficulties in adhering to complex pediatric treatment regimens, underscoring a vulnerable clinical scenario where caregiver affective distress and perceived well-being warrant integrated assessment. Consequently, pediatric healthcare settings should prioritize routine dual-screening for both depressive symptoms and quality of life to identify at-risk families and implement comprehensive support strategies.

## 5. Conclusions

The results obtained demonstrate that elevated depressive symptomatology is strongly associated with a decrease in quality of life across all domains among family caregivers of dependent children. These findings highlight the importance of healthcare professional interventions aimed at screening, preventing, and alleviating depressive symptoms to safeguard caregiver well-being. Future research should prioritize prospective longitudinal designs to clarify the causal pathways between depressive symptoms and quality of life over time.

## Figures and Tables

**Table 1:** Search strategy.

Data Base	Chain Used
PubMed	(Quality of Life[mj] OR quality of life[tiab] OR life quality[tiab] OR QoL[tiab]) AND (Depression[mj] OR Depress*[tiab]) AND (Caregivers[mj] OR caregiv*[tiab] OR care giv*[tiab] OR carer*[tiab])
CINAHL	(MM Quality of Life OR AB quality of life OR AB life quality) AND (MM Depression OR AB Depress*) AND (MM Caregivers OR AB caregiv* OR AB care giv* OR AB carer*)
PsycInfo	(MJSUB(Quality of Life) OR AB(quality of life) OR AB(life quality) OR AB(QoL)) AND (MJSUB(Depression) OR AB(Depressive Symptoms) OR AB(Depress*)) AND (MJSUB(Caregivers) OR AB(Caregiv*) OR AB(carer*))
Scopus	(INDEXTERMS(“Quality of Life”) OR TITLE-ABS-KEY(“quality of life”) OR TITLE-ABS-KEY(“life quality”)) AND (INDEXTERMS(“Depression”) OR TITLE-ABS-KEY(“depress*”)) AND (INDEXTERMS(“caregivers”) OR TITLE-ABS-KEY(“caregiv*”) OR TITLE-ABS-KEY(“care giv*”) OR TITLE-ABS-KEY(“carer*”))

**Table 2:** Description of the studies included in the review.

Author–Year	Country	Design	N	Recipients of Care	Caregiver Characteristics	Measuring Depressive Symptoms	Measuring Quality of Life
Albayrak, 2019 [26]	Türkiye	DT	101	Neuromotor–Neuromuscular Imp	Mother	BDI	SF-36
Al-Dwaikat, 2025 [27]	Jordan	DT	80	ASD	Both	DASS-21	WHOQOL-BREF
Atak, 2026 [28]	Türkiye	DT	40	Neuromotor–Neuromuscular Imp	Mother	BDI	SF-36
Doly, 2024 [29]	Bangladesh	DT	150	Neuromotor–Neuromuscular Imp	Both	PHQ-9	WHOQOL-BREF
Dong, 2026 [30]	China	DT	217	Sd. Down	Others	HAMD-17	WHOQOL-BREF
Driscoll, 2009 [31]	USA	DT	122	Chronic Dis	Both	CES-D	CQOLCF
Favero, 2010 [24]	Brazil	DT	20	ASD	Mother	BDI-II	WHOQOL-BREF
Fullwood, 2025 [32]	Australia	DT	26	Sd. Down	Others	DASS	WHOQOL-BREF
Jamali, 2020 [33]	Iran	DT	203	Neuromotor–Neuromuscular Imp	Mother	BDI-II	WHOQOL-BREF
Joung, 2022 [34]	Republic of Korea	DT	154	Sd. Down	Mother	K-DAS	WHOQOL-BREF
Kim, 2025 [35]	Republic of Korea	DT	162	Neuromotor–Neuromuscular Imp	Both	CES-D	WHOQOL
Kokui, 2016 [36]	Ghana (Africa)	DT	130	Neuromotor–Neuromuscular Imp	Others	DASS-21	WHOQOL-BREF
Konowalek, 2025 [37]	Poland	DT	372	ASD	Both	BDI	WHOQOL
Koyuncu, 2025 [38]	Türkiye	DT	42	Neuromotor–Neuromuscular Imp	Both	HADS	WHOQOL-BREF
López Márquez, 2014 [39]	Mexico	DT	31	Neuromotor–Neuromuscular Impr	Both	BDI-II	SF-36
Loutou, 2025 [40]	Canada	DT	50	Neuromotor–Neuromuscular Imp	Both	PHQ-9	SF-12
Mahmoudi-Gharaei, 2011 [41]	Iran	DT	49	Chronic Dis	Both	DASS	WHOQOL-BREF
Medrano, 2013 [25]	USA	DT	579	Chronic Dis	Both	HADS	HRQOL
Ones, 2005 [42]	Türkiye	DT	46	Neuromotor–Neuromuscular Imp	Mother	BDI-II	NHP-1
Payakachat, 2011 [43]	USA	DT	65	Neuromotor–Neuromuscular Imp	Both	CES-D	SF-6D
Piovensan, 2015 [44]	Brazil	DT	40	ASD	Mother	BDI	WHOQOL-BREF
Reed, 2022 [45]	Canada	DT	99	Chronic Dis	Both	CES-D	SF-36
Restrepo, 2023 [46]	Colombia	DT	132	ASD	Others	DASS-21	SF-36
Santo, 2011 [47]	Brazil	DT	32	Chronic Dis	Both	BDI	SF-36
Sarajlija, 2013 [48]	Serbia	DT	49	Neuromotor–Neuromuscular Imp	Mother	BDI-II	SF-36
Shaikan, 2020 [49]	Qatar	DT	330	Chronic Dis	Both	BDI	PACQLQ
Sonune, 2021 [50]	India	DT	203	Neuromotor–Neuromuscular Imp	Mother	MADRS	WHOQOL-BREF
Stock, 2025 [23]	United Kingdom	MRL	525	Chronic Dis	Both	HASD	PedsQL
Taha, 2021 [51]	USA	DT	58	Neuromotor–Neuromuscular Imp	Both	PHQ-9	CQLI-R
Tekinarslan, 2013 [52]	Türkiye	DT	252	ASD, Cerebral Palsy and Sd. Down	Mother	BDI-II	WHOQOL-BREF–TR
Toledano, 2020 [53]	Mexico	DT	416	Chronic Dis	Both	BDI	WHOQOL
Wang, 2021 [54]	China	DT	79	ASD	Both	DASS-21	Beach Center FQOL Scale
Willems, 2021 [55]	Germany	DT	184	Neuromotor–Neuromuscular Imp	Both	BDI	EQ-5D

● Abbreviations: DT (Descriptive Cross-Sectional); ASD (Autism Spectrum Disorder); MRL: Longitudinal Repeated Measures; Sd. Down: Down syndrome; Chronic Dis: chronic diseases; Neuromotor–Neuromuscular Imp: neuromotor–neuromuscular impairment. ● Depression scales: BDI (Beck Depression Inventory); BDI-II (Beck Depression Inventory—Second Edition); DASS (Depression, Anxiety and Stress Scale); DASS-21 (Depression, Anxiety and Stress Scale—21 Items); PHQ-9 (Patient Health Questionnaire—9); CES-D (Center for Epidemiological Studies Depression Scale); HADS (Hospital Anxiety and Depression Scale); K-DAS (Korean Depression and Anxiety Scales); MADRS (Montgomery–Asberg Depression Rating Scale); HAMD-17 (Hamilton Depression Rating Scale). ● Quality of life scales: WHOQOL (World Health Organization Quality of Life); WHOQOL-BREF (World Health Organization Quality of Life—Abbreviated version); WHOQOL-BREF–TR (World Health Organization Quality of Life—Abbreviated version, Turkish version); SF-36 (36-Item Short Form Survey); SF-12 (12-Item Short Form Survey); SF-6D (Short Form 6 Dimension); CQOLCF (Caregiver Quality of Life Index-Cancer); NHP-1 (Nottingham Health Profile—Part 1); PACQLQ (Pediatric Asthma Caregiver’s Quality of Life Questionnaire); CQLI-R (Quality of Life Index—Revised); Beach Center FQOL Scale (Beach Center Family Quality of Life Scale); EQ-5D (EuroQol—5 Dimension); HRQOL (Health Related Quality of Life); PedsQL (Pediatric Quality of Life Inventory Family).

**Table 3:** Description of risk of bias of the included studies.

Author–Year	Selection	Classification	Confounding
Albayrak, 2019a [26] MCS	−	+	+
Albayrak, 2019b [26] PCS	−	+	+
Al-Dwaikat, 2025 [27]	−	+	−
Atak, 2026 [28]	−	+	−
Doly, 2024 [29]	−	+	−
Dong, 2026 [30]	−	+	?
Driscoll, 2009a [31] Mother Subgroup	−	+	+
Driscoll, 2009b [31] Father Subgroup	−	+	+
Favero, 2010 [24]	−	+	−
Fullwood, 2025 [32]	−	+	−
Jamali, 2020 [33]	−	+	−
Joung, 2022 [34]	−	+	−
Kim, 2025 [35]	−	+	−
Kokui, 2016 [36]	−	+	−
Konowalek, 2025 [37]	−	+	−
Koyuncu,2025 [38]	−	+	−
López Márquez, 2014 [39]	−	+	−
Loutou, 2025 [40]	−	+	−
Mahmoudi-Gharaei, 2011 [41]	−	+	−
Medrano, 2013 [25]	−	+	−
Ones, 2005 [42]	−	+	−
Payakachat, 2011 [43]	−	+	−
Piovensan, 2015 [44]	−	+	?
Reed, 2022 [45]	−	+	−
Restrepo, 2023 [46]	−	+	−
Santo, 2011 [47]	−	+	−
Sarajlija, 2013 [48]	−	+	−
Shaikan, 2020 [49]	−	+	−
Sonune, 2021 [50]	−	+	−
Stock, 2025 [23]	−	+	?
Taha, 2021 [51]	−	+	+
Tekinarslan, 2013 [52]	−	+	−
Toledano,2020 [53]	−	+	?
Wang, 2021 [54]	−	+	−
Willems, 2021 [55]	−	+	−

Key: (−) risk of bias; (+) low risk of bias; (?) not enough information to evaluate.

**Table 4:** Results of the meta-analysis covering the relationship between depressive symptoms and quality of life.

	Global/	Whole Sample/Subgroups					Combined Effect			Heterogeneity			Sensitivity		Publication Bias			
Dependent Variable	Dimensions			K	N	Average N	r	Lower Limit	Upper Limit	Q (df)	*p*	I^2^	Analyses		Funnel	Egger’s	Trim and Fill	
		Criteria	Categories										R Max	%		*p*-Value	r	%
Quality of Life	Global	Whole sample	--	4238	26	163.00	−0.532	−0.599	−0.458	27.62 (25)	0.32	9.51	−0.532	0.00	Asym	0.73	−0.532	0.00
		Type of care-recipient	ASD	843	6	140.5	−0.599	−0.706	−0.465	5.39 (5)	0.36	7.26	−0.599	0.00	Asym	NV	−0.573	4.27
			Sd. Down	397	3	132.33	−0.456	−0.708	−101	1.30 (2)	0.52	0.00	−0.456	0.00	NV	NV	−0.456	0.00
			Chronic Dis.	1838	6	306.33	−0.527	−0.605	−0.438	6.44 (5)	0.26	22.39	−0.527	0.00	Asym	NV	−0.527	0.00
			Neuromotor–Neuromuscular Imp.	1264	11	114.91	−0.517	−0.652	−0.350	9.28 (10)	0.50	0.00	−0.517	0.00	Asym	0.85	−0.517	0.00
		Control of confounders	No	4105	30	136.83	−0.541	−0.608	−0.467	25.79 (22)	0.18	14.72	−0.541	0.00	Asym	0.68	−0.541	0.00
			Yes	476	5	95.2	−0.465	−0.738	−0.060	1.47 (2)	0.47	0.00	−0.465	0.00	NV	NV	−0.465	0.00
	SF-36 (PCS)	Whole sample	--	503	7	71.86	−0.544	−0.727	−0.289	6.75 (6)	0.34	11.21	−0.544	0.00	Asym	NV	−0.544	0.00
	SF-36 (MCS)	Whole sample	--	503	7	71.86	−0.537	−0.717	−0.290	5.91 (6)	0.43	0.00	−0.537	0.00	Asym	NV	−0.537	0.00
	Environmental	Whole sample	--	1282	11	116.54	−0.410	−0.518	−0.289	7.28 (10)	0.69	0.00	−0.410	0.00	Asym	0.49	−0.32	21.98
	Physics	Whole sample	--	1282	11	116.54	−0.481	−0.587	−0.358	5.93 (10)	0.82	0.00	−0.481	0.00	Asym	0.97	−0.42	11.89
	Psychological	Whole sample	--	1282	11	116.54	−0.527	−0.635	−0.399	8.10 (10)	0.61	0.00	−0.527	0.00	Asym	0.44	−0.49	7.12
	Social	Whole sample	--	1262	10	126.2	−0.486	−0.604	−0.347	5.53 (9)	0.78	0.00	−0.486	0.00	Asym	0.67	−0.48	0.00

Abbreviations: K: number of studies; N: sample size; R: combined correlation coefficient; r max: maximum value of the combined effect for sensitivity analysis eliminating one study at a time; %: percentage of variation from the original combined effect; ASD: autism spectrum disorder; Sd. Down: Down syndrome; Chronic Dis: chronic diseases; Neuromotor–Neuromuscular Imp: neuromotor–neuromuscular impairment; Asym: asymmetric; NV: not valuable (its assessment is not recommended when there are few studies).

## Data Availability

No new data were created or analyzed in this study.

## Supplementary Materials

The following supporting information can be downloaded at https://www.mdpi.com/article/10.3390/healthcare14172765/s1, Supplementary Material S1: GRADE table. Supplementary Material S2: Results of the meta-analysis.
