# Supplementary material for: Relationship Between Depressive Symptoms and Quality of Life in Family Caregivers of Dependent Children: A Systematic Review with Meta-Analysis
Source: Healthcare (Basel). 2026 Sep 1;14(17):2765. doi: 10.3390/healthcare14172765 (PMC13565090; doi:10.3390/healthcare14172765)
Supplement: Supplementary file 1 [file healthcare-14-02765-s001.zip › healthcare-4453579-supplementary.pdf]

Supplementary material S1, GRADE Table

Outcome / Variable	Number of studies (K)	Total sample (N)	Risk of bias / Confounders	Inconsistency (I2)	Indirectness	Imprecision (Sample / CI)	Publication bias (Egger / Funnel)	Quality of evidence (GRADE)
Quality of life global (Whole sample)	26	4238	Partially controlled (K=5 yes / K=30 no)	Very low (I2 = 9.51%)	Not serious	Not serious (N > 300, narrow CI)	No significant bias (p = .73)	Low
SF-36 Physical Component Summary (PCS)	7	503	Serious	Very low (I2 = 11.21%)	Not serious	Moderate (N < 100 per study)	Not evaluable / Asymmetric	Low
SF-36 Mental Component Summary (MCS)	7	503	Serious	Null (I2 = 0.00%)	Not serious	Moderate (N < 100 per study)	Not evaluable / Asymmetric	Low
Environmental domain	11	1282	Serious	Null (I2 = 0.00%)	Not serious	Not serious (N > 300)	No significant bias (p = .49)	Low
Physical domain	11	1282	Serious	Null (I2 = 0.00%)	Not serious	Not serious (N > 300)	No significant bias (p = .97)	Low
Psychological domain	11	1282	Serious	Null (I2 = 0.00%)	Not serious	Not serious (N > 300)	No significant bias (p = .44)	Low
Social domain	10	1262	Serious	Null (I2 = 0.00%)	Not serious	Not serious (N > 300)	No significant bias (p = .67)	Low

Downgraded due to serious risk of bias (cross-sectional design, non-probability sampling, and unadjusted residual confounding in primary studies).

Supplementary Material S2, results of the meta-analysis

Variable dependiente	Global/ Dimensiones	Muestra total		N	K	Promedio N	Efecto combinado			Heterogeneidad			Sensibilidad Análisis		Sesgo de publicación			
		Criterio	Categorías				r	Límite inferior	Límite superior	Q (df)	Valor p	I^2	R máx	%	Embudo	Egger's Valor p	Trim & Fill r	%
Calidad de vida	Global	Muestra total	--	4238	26	163,00	-.532	-.599	-.458	27.62 (25)	0.32	9.51	-.532	0.00	Asimétrico	,73	-.532	0.00
		Patología cuidado	TEA	843	6	140,5	-.599	.-706	-.465	5.39 (5)	0.36	7.26	-.599	0.00	Asimétrico	No procede	-.573	4.27
			Sd. Down	397	3	132,33	-.456	-.708	-.101	1.30 (2)	0.52	0.00	-.456	0.00	No procede	No procede	-.456	0.00
			Enf. Crónica	1838	6	306,33	-.527	-.605	-.438	6.44 (5)	0.26	22.39	-.527	0.00	Asimétrico	No procede	-.527	0.00
			Af. Neuromotor-Neuromuscular	1264	11	114,91	-.517	-.652	-.350	9.28 (10)	0.50	0.00	-.517	0.00	Asimétrico	.85	-.517	0.00
		Control de Factores de confusión	No	4105	30	136,83	-.541	-.608	-.467	25.79 (22)	0.18	14.72	-.541	0.00	Asimétrico	.68	-.541	0.00

			Si	476	5	95,2	-.465	-.738	-.060	1.47 (2)	0.47	0.00	-.465	0.00	No procede	No procede	-.465	0.00
	SF- 36 (PCS)	Muestra entera	--	503	7	71,86	-.544	-.727	-.289	6.75 (6)	0.34	11.21	-.544	0.00	Asimétrico	No procede	-.544	0.00
	SF- 36 (MCS)	Muestra entera	--	503	7	71,86	-.537	-.717	-.290	5.91 (6)	0.43	0.00	-.537	0.00	Asimétrico	No procede	-.537	0.00
	Ambiente	Muestra entera	--	1282	11	116.54	-.410	-.518	-.289	7.28 (10)	0.69	0.00	-.410	0.00	Asimétrico	.49	-.32	21.98
	Física	Muestra entera	--	1282	11	116.54	-.481	-.587	-.358	5.93 (10)	0.82	0.00	-.481	0.00	Asimétrico	.97	-.42	11.89
	Psicológica	Muestra entera	--	1282	11	116.54	-.527	-.635	-.399	8.10 (10)	0.61	0.00	-.527	0.00	Asimétrico	.44	-.49	7.12
	Social	Muestra entera	--	1262	10	126.2	-.486	-.604	-.347	5.53 (9)	0.78	0.00	-.486	0.00	Asimétrico	.67	-.48	0.00
